# A marine and salt marsh sediment organic carbon database for European regional seas (EURO-CARBON)

**DOI:** 10.1016/j.dib.2025.111595

**Published:** 2025-05-03

**Authors:** Anna Elizabeth Løvgren Graversen, Christian Lønborg, Anna Maria Addamo, Sidsel Gurholt Pedersen, Silvia Chemello, Irene Alejo, Eugenia T. Apostolaki, Maria E. Asplund, William E.N. Austin, Dimitar Berov, Daniela Berto, Mats Björk, Kirsty Black, Nikola Bobchev, Stefano Bonaglia, Gunhild Borgersen, Tjeerd Bouma, Mark J. Costello, Martin Dahl, Elena Diaz-Almela, Panagiotis D. Dimitriou, Carlos M. Duarte, Carmen Leiva Dueñas, Pavlos T. Efthymiadis, Ines Mazarrasa Elosegui, Maria Recio Espinosa, Helena L. Filipsson, Marcos Fontela, Stein Fredriksen, Helene Frigstad, Karine Gagnon, Catalina A. Garcia-Escudero, Michele Giani, Anne Grouhel-Pellouin, Roberta Guerra, Martin Gullström, Hege Gundersen, Kasper Hancke, Claudia Majtényi-Hill, Corallie Hunt, Karina Inostroza, Ioannis Karakassis, Ventzislav Karamfilov, Stefania Klayn, Katarzyna Koziorowska, Karol Kuliński, Paul Lavery, Wytze K. Lenstra, Ana I. Lillebø, Ella Logemann, Paolo Magni, Núria Marbà, Candela Marco-Mendez, Marcio Martins, Miguel Angel Mateo, Briac Monnier, Peter Mueller, Joao M. Neto, Nafsika Papageorgiou, Carlos Eduardo de Rezende, Juan Carlos Farias Pardo, Jose Antonio Juanes De La Peña, Gérard Pergent, Nerea Piñeiro-Juncal, Joanne Preston, Federico Rampazzo, Gloria Reithmaier, Thorsten B.H. Reusch, Sarah Reynolds, Aurora M. Ricart, Rui Santos, Carmen B. de los Santos, Isaac R. Santos, Eduard Serrano, Oscar Serrano, Caroline P. Slomp, Craig Smeaton, Montserrar Soler, Ana I. Sousa, Timo Spiegel, Angela Stevenson, Jonas Thormar, Hilde Cecilie Trannum, Niels A.G.M. van Helmond, Sarah Paradis, Salvatrice Vizzini, Emma A. Ward, Yvonne Y.Y. Yau, Rym Zakhama-Sraieb, Imen Zribi, Olga M. Zygadlowska, Dorte Krause Jensen

**Affiliations:** a Department of Ecoscience, Aarhus University, Aarhus, Denmark; b Department of Ecoscience, Aarhus University, Roskilde, Denmark; c Faculty of Biosciences and Aquaculture, Nord University, Bodø, Norway; d CIIMAR—Interdisciplinary Centre of Marine and Environmental Research, University of Porto, 4450-208 Matosinhos, Portugal; e Marine Research Center (CIM-XM3), University of Vigo, Lagoas Marcosende S/N, 36310 Vigo, Spain; f Institute of Oceanography, Hellenic Centre for Marine Research, Heraklion, Crete, Greece; g Department of Biological and Environmental Sciences, University of Gothenburg, Gothenburg, Sweden; h University of St Andrews, School of Geography & Sustainable Development, St Andrews, United Kingdom; i Scottish Association for Marine Science, Dunstaffnage, Argyll, Scotland, United Kingdom; j IBER-BAS, Institute of Biodiversity and Ecosystem Research at the Bulgarian Academy of Sciences, Sofia, Bulgaria; k ISPRA The Italian Institute for Environmental Protection and Research, Chioggia, Italy; l Stockholm University, Department of Ecology, Environment and Plant Sciences, Stockholm, Sweden; m RSK Group, Marine Team International Projects Group, Stirling, Scotland, United Kingdom; n University of Gothenburg, Department of Marine Sciences, Gothenburg, Sweden; o NIVA, Norwegian institute for Water research, Oslo, Norway; p NIOZ, Royal Netherlands Institute for Sea Research, Yerseke, the Netherlands; q Södertörn university, Natural Sciences, Technology and Environmental Studies, Sweden; r Grupo Tragsa, Madrid, Spain; s Department of Biology, University of Crete, Heraklion, Crete, Greece; t King Abdullah University of Science and Technology (KAUST), Saudi Arabia; u IHCantabria-Instituto de Hidráulica Ambiental de la Universidad de Cantabria, Parque Científico y Tecnológico de Cantabria, Santander, Spain; v Lund University, Department of Geology, Lund, Sweden; w Instituto de Investigaciones Marinas (IIM‐CSIC), Vigo, Spain; x University of Oslo, Department of Biosciences, Oslo, Norway; y IMR, Institute of Marine Research, Flødevigen Research Station, His, Norway; z National Institute of Oceanography and Applied Geophysics (OGS), Trieste, Italy; aa IFREMER - French Institute for Ocean Science, Plouzané, France; ab University of Bologna, Department of Physics and Astronomy “Augusto Righi” - DIFA, Italy; ac NatureScot, Great Glen House, Leachkin Road, Inverness, IV3 8NV, Scotland, United Kingdom; ad BIOSFERA Environmental Education, Research and Conservation, L'Hospitalet de Llobregat, Barcelona, Spain; ae Institute of Oceanology Polish Academy of Sciences, Sopot, Poland; af Edith Cowan University, Joondalup, Centre for Marine Ecosystems Research, Australia; ag Radboud University, Radboud Institute for Biological and Environmental Sciences, Department of Microbiology, Heyendaalseweg 135, 6525 AJ Nijmegen, the Netherlands; ah ECOMARE, CESAM—Centre for Environmental and Marine Studies, Department of Biology, University of Aveiro, 3810-193 Aveiro, Portugal; ai University of Hamburg, Institute of Plant Science and Microbiology, Applied Plant Ecology, Hamburg, Germany; aj CNR-IAS, Consiglio Nazionale delle Ricerche, Istituto per lo studio degli impatti Antropici e Sostenibilità in ambiente marino, Loc. Sa Mardini, Torregrande, 09170, Oristano, Italy; ak Mediterranean Institute for Advanced Studies (CSIC-IMEDEA), Spain; al CEAB-CSIC, Centre d'Estudis Avançats de Blanes, Consejo Superior de Investigaciones Científicas, Blanes, Spain; am Centre of Marine Sciences (CCMAR/CIMAR LA), Campus de Gambelas, Universidade do Algarve, Faro, Portugal; an University of Corsica, UMR CNRS SPE 6134, Campus Grimaldi, France; ao University of Corsica, UAR CNRS 3514 STELLA MARE, Plateforme Stella Mare, France; ap RPTU Kaiserslautern-Landau, Germany; aq MARE - Marine and Environmental Sciences Centre / ARNET - Aquatic Research Network, University of Coimbra, Coimbra, Portugal; ar National and Kapodistrian University of Athens, Department of Agricultural Development, Agri-Food and Natural Resources Management, Greece; as UENF, Universidade Estadual do Norte Fluminense, Environmental Sciences Laboratory, Rio de Janeiro, Brazil; at University of Agder (UiA) and Norwegian institute for Water research, Oslo, Norway; au University of Portsmouth, School of Environment & Life Sciences, Portsmouth, United Kingdom; av GEOMAR, Helmholtz-Centre for Ocean Research, Kiel, Germany; aw Centro de Estudios Avanzados de Blanes (CSIC-CEAB), Spain; ax Department of Earth Sciences, Utrecht University, Princetonlaan 8a, 3584 CB Utrecht, the Netherlands; ay Geological Institute, Department of Earth and Planetary Sciences, ETH Zürich, Switzerland; az Department of Earth and Marine Sciences, University of Palermo, Local Research Unit of CoNISMa, Palermo, Italy; ba University of Manouba, High Institute of Biotechnology of Sidi Thabet, BiotechPôle, BP-66, 2020 Sidi Thabet, Ariana, Tunisia; bb University of Tunis El Manar, Faculty of Sciences of Tunis, Laboratory of Diversity, Management and Conservation of Biological Systems, LR18ES06, Tunis, Tunisia; bc Instituto de Ciencias del Mar, Consejo Superior de Investigaciones Científicas (ICM-CSIC), Barcelona, Spain; bd GEOMAR Helmholtz-Centre for Ocean Research Kiel, Marine Evolutioary Ecology, Kiel, Germany; be Istituto Centrale per la Ricerca scientifica e tecnologica Applicata al Mare (ICRAM), Chioggia, Italy

**Keywords:** Sediment organic carbon, Blue carbon, Marine sediments, Salt marsh, Seagrass

## Abstract

Marine and salt marsh sediments contain large amounts of organic carbon (OC) and are therefore important in the global carbon cycle. Here, we collated previously published and unpublished measurements of sediment OC in marine and salt marsh sediments in European regional seas (EURO-CARBON; available at https://doi.org/10.5281/zenodo.14905489). To the extent possible the OC data were complemented by variables such as sediment porosity and dry bulk density. The EURO-CARBON dataset holds 61306 individual data entries of sediment OC content from different regions of European regional seas. Around three quarters (76%) were collected in coastal and deep sea bare sediments, 18% from salt marshes, 7% from seagrass habitats, and 0.03% from macroalgal habitats. For all habitats and sediment depth layers the OC content varied between <0.1 and 41.56 % (avg.: 2.47 ± 3.37 %; median: 1.39 %), with the content generally decreasing in the following sequence: salt marsh (5.01 ± 5.96 %; 3.03 %) > seagrass (2.37 ± 5.96 %; 3.03 %) > bare sediment (1.88 ± 2.03 %; 1.20 %). The EURO-CARBON dataset will serve as a basis for future work, and it will be an important resource for researchers, managers, and policymakers working towards protecting sediment OC pools.

Specifications Table

**Table:** 

Subject	Earth & Environmental Sciences
Specific subject area	Marine and salt marsh sediments contain large amounts of organic carbon (OC) and are therefore important in the global carbon cycle. Here, we collated previously published and unpublished measurements of sediment OC in marine and salt marsh sediments in European regional seas.
Type of data	The data compiled have been deposited in an open-access repository under the following link: https://doi.org/10.5281/zenodo.14905489. The file can be downloaded as a *.csv merged file.
Data collection	Initially the research community was invited through a public call to contribute data to establish a database of OC and related variables in marine sediments. Secondly, data were retrieved from public databases. Thirdly, a detailed search was performed in Google Scholar. Further searches were conducted in the reference lists of the identified studies. Additional studies were included from existing reviews on sediment OC and finally, we included data from MSc or PhD theses, and other published reports based on our knowledge of the research field.
Data source location	The data were collected in coastal and deep-sea settings within European Regional Seas, which here includes the Baltic Sea, the Black Sea, the North-east Atlantic Ocean, and the Mediterranean Sea.
Data accessibility	Repository name: Zenodo open data repository (CERN) Data identification number: doi: 10.5281/zenodo.14905489 Direct URL to data: https://doi.org/10.5281/zenodo.14905489
Related research article	A subset of the dataset has been used separately in different publications. However, the current dataset is the first time to combine these datasets with unpublished data to provide a more comprehensive database for European Regional seas.

## 1 Value of the Data

• The compilation contains sediment organic carbon data collected across European regional seas.
• The dataset is an important resource for researchers, managers, and policymakers working towards protecting sediment organic carbon pools.
• The compilation can be used to assess carbon storage and the sensitivity to anthropogenic pressures.

## 2 Background

Marine and salt marsh sediments are some of the major organic carbon (OC) reservoirs on the planet and are therefore vital components of the global carbon cycle [1]. Recent estimates suggest that globally the top 5 cm of surface marine sediments alone contain an OC reservoir of around 87,000 ± 43,000 Mt, while in the top 1 m of these sediments the pool may be as large as 2.3 million Mt OC [1]. However, the size and residence time of sedimentary OC stocks vary considerably with geological, physical, chemical, and biological settings and also depend on the temporal and spatial scales under consideration [1]. While the capacity of marine sediments to preserve OC has intrigued biogeochemists for decades [2], it is only more recently that this subject has gained considerable attention within the wider scientific community. This attention has focused around “Blue Carbon” which are all biologically driven carbon fluxes and storage in marine systems that are amenable to management. Blue Carbon research has grown rapidly over the past decade, where the focus has been on quantifying OC stocks, managing and protecting organic carbon-rich habitats and potentially increasing their capacity to capture carbon dioxide and retain OC [3]. However, understanding OC fluxes and preservation processes and providing potential management inputs require reliable data on the location of important habitats, OC content, OC stocks and OC accumulation rates along with site-specific physical and biogeochemical conditions.

Datasets exist especially for OC content but is more limited for OC stocks and accumulation rates. These data can, if compiled and standardized, provide a powerful resource for scientists to deliver new OC distribution maps, perspectives on the distribution pattern and a better understanding of controlling factors over larger spatial and temporal scales. Also, standardized datasets can help identify geographical areas and habitats with limited sampling efforts needing complementary data collection, and also highlight avenues for future research. This data report aims to provide the scientific community with a comprehensive compilation of sediment OC contents, associated sediment variables, and environmental conditions in European regional seas, the EURO-CARBON database. This compilation includes data available in public repositories and scientific papers, but also currently unpublished datasets. In some instances, the EURO-CARBON database also includes above and below ground biomass data for associated vegetated coastal ecosystems such as seagrasses and salt marshes. As per the nature of a data report manuscript, only a preliminary discussion of the included data is presented together with some possible future uses of the dataset.

## 3 Data Description

The data included in the EURO-CARBON database originate from multiple sources and therefore different research groups have been involved in the sample collection, analysis, and/or collecting associated information. Quality assurance and quality control (QA/QC) of large datasets, such as EURO-CARBON, is critical to ensure that included data are trustworthy and useful. Therefore, we have not included data that were considered of “low-quality”. Nevertheless, a degree of variability within the dataset was accepted given that multiple groups and laboratories were involved in the data compilation.

Obvious errors, such as incorrect geographical coordinates, were corrected, while errors that could not be resolved, such as unrealistically high values, were excluded from the dataset. Prior to excluding suspected erroneous observations, where possible, data originators were directly contacted to seek confirmation of the observations. During these steps, excessively narrow standards, known as “data grooming”, were avoided so potential real patterns could be identified. Given the wide range of environmental conditions in marine sediments, influenced by factors such as local anthropogenic activities, establishing reliable lower and upper limits for sediment OC content is challenging due to their inherent variability. Initially data plots were used to identify potential outliers within the different habitats such as extreme low or high OC contents. Lower limits of OC contents are difficult to establish and in cases where concentrations were below the detection limit (around 0.1%), zero values were replaced with half the value of the limit of detection. Additionally, upper OC contents for surface sediments previously reported in the literature were used. These include those reported in systems such as river deltas including the Fly river delta (up to 2.5%; ([4]), fjords (up to 8.8%; [5]) and sediments dominated by seagrass (up to 19.8%;([6]) or microphytobenthos (up to 13.7%; [7]). In open ocean systems, OC contents up to 2.3% have been reported in the literature in Hadal trenches which are considered “OC hotspots” [8]. In salt marshes levels above 40% OC have been detected in some areas [9].

Overall, the lower and upper limits for OC contents were used in EURO-CARBON as general guiding limits for identifying potential errors in observations. Once identified, potential errors were either corrected by the data originator, or if not possible (e.g., values showing signs of contamination) these were excluded from the database. Similar approaches, using previously published values to identify potential errors, were used for other variables included in EURO-CARBON, for example δ^13^C, which reflects varying degrees of terrestrial (from −22 ‰ to −30 ‰) and marine (−10 ‰ to −31 ‰) OC sources, can vary substantially [10].

In EURO-CARBON, a total of 61306 data entries for OC content were included, with the following distribution: 76% from bare sediments, 18% from salt marshes, 7% from seagrass habitats, and 0.03% from macroalgal habitats (Table 2). For all sediment depths and habitats, the OC content varied between <0.1 and 41.56 % (mean: 2.46 ± 3.36 %; median: 1.39 %), with the average contents decreasing in the following sequence: salt marsh (5.01 ± 5.96 %; 3.03 %) > seagrass (2.37 ± 5.96 %; 1.11%) > algal habitat (1.98 ± 1.23 %; 2.16 %) > bare sediment (1.88 ± 2.03 %; 1.20 %). In addition, comparing the averages and medians of surface and deeper sediment layers, we found generally higher contents in the surface layers (Table 2).

**Fig. 1:**
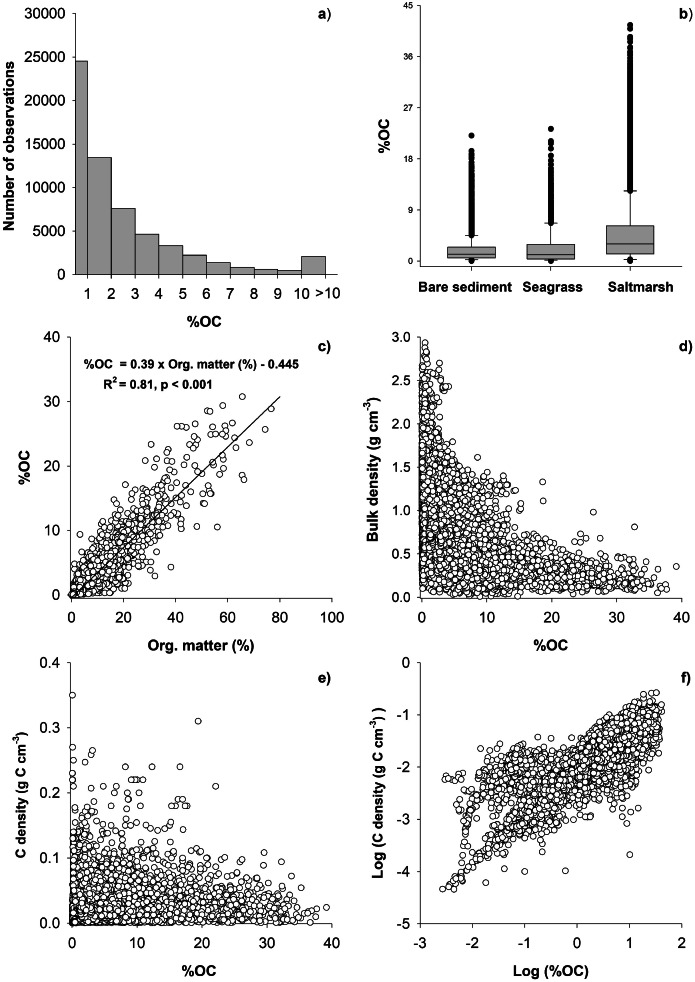
Histogram (a) showing the distribution of all percent organic carbon content (%OC) observations included in the EURO-CARBON database and box plot (b) of %OC in bare, seagrass and salt marsh sediments. Relationships (c, d) between %OC and the % organic matter (org. matter (%)) and dry bulk density (DBD (g cm^−3^)), respectively, are also shown. The relationship between carbon density (g C cm^−3^) and %OC are shown both for raw (e) and log transformed (f) values. The lines in the box plot (b) represent median values, the limits of the boxes represent 25-75 percentiles, and the whiskers the data range. Please note that data from macroalgae habitats are not shown in (b) as these data were only collected in one location.

The dataset showed that within the different habitats, there was a large variation in the %OC content, with the coefficient of variation (CV, i.e., dispersion of the data around the mean) being highest in seagrass sediments followed by saltmarshes and bare sediments (Table 2). The variability in OC content is likely due to differences in local physical and biogeochemical conditions, and different degree of organic enrichment. Also, some studies (such as regions along the Norwegian coast) had a bias towards locations impacted by eutrophication as these sediment samples were collected in connection with recipient surveys (incl. screening for municipal wastewater impacts). This means that these had higher OC-values than at reference coastal locations. A large part (40 %) of the OC content data were below 1 % and these were predominantly collected in bare sediments, while the highest contents were generally found in salt marsh sediments (Fig. 1 and s1).

In the EURO-CARBON database, multiple variables besides %OC were included; however, these were not available in all instances (see Table s2 and s3 for summaries). For example, the % organic matter (loss on ignition-LOI) content and dry bulk density (DBD) were measured in 19 % and 30 % of the included observations, respectively (Table s2 and s3). In the EURO-CARBON database, we only included measurements which have directly determined the OC content (e.g. elemental analysers), and excluded OC estimates only obtained, by the LOI technique. We are aware that the significant relationship between %OC and Org. matter (%) (LOI) across habitats (Fig. 1) should be taken with caution as the OM content measured by LOI depend on factors such as the sediment composition (e.g. clay and salt content) [11], carbonate content, ignition time and temperature [12].. Overall, the %OC content declined with the DBD (Fig. 1). Plotting the %OC against OC density showed large variability, while the log-log plot showed a near linear fit, though still with large variability (Fig. 1). In addition, OC accumulation rates were only measured in 1 % (720 estimates) of the included measurements (Table s3). The %OC content of the above- and below-ground biomass of the vegetated habitats included 984 data entries (82% for saltmarsh and 18% for seagrass), while biomass estimates were measured in 693 samples (Table s4).

**Fig. 2:**
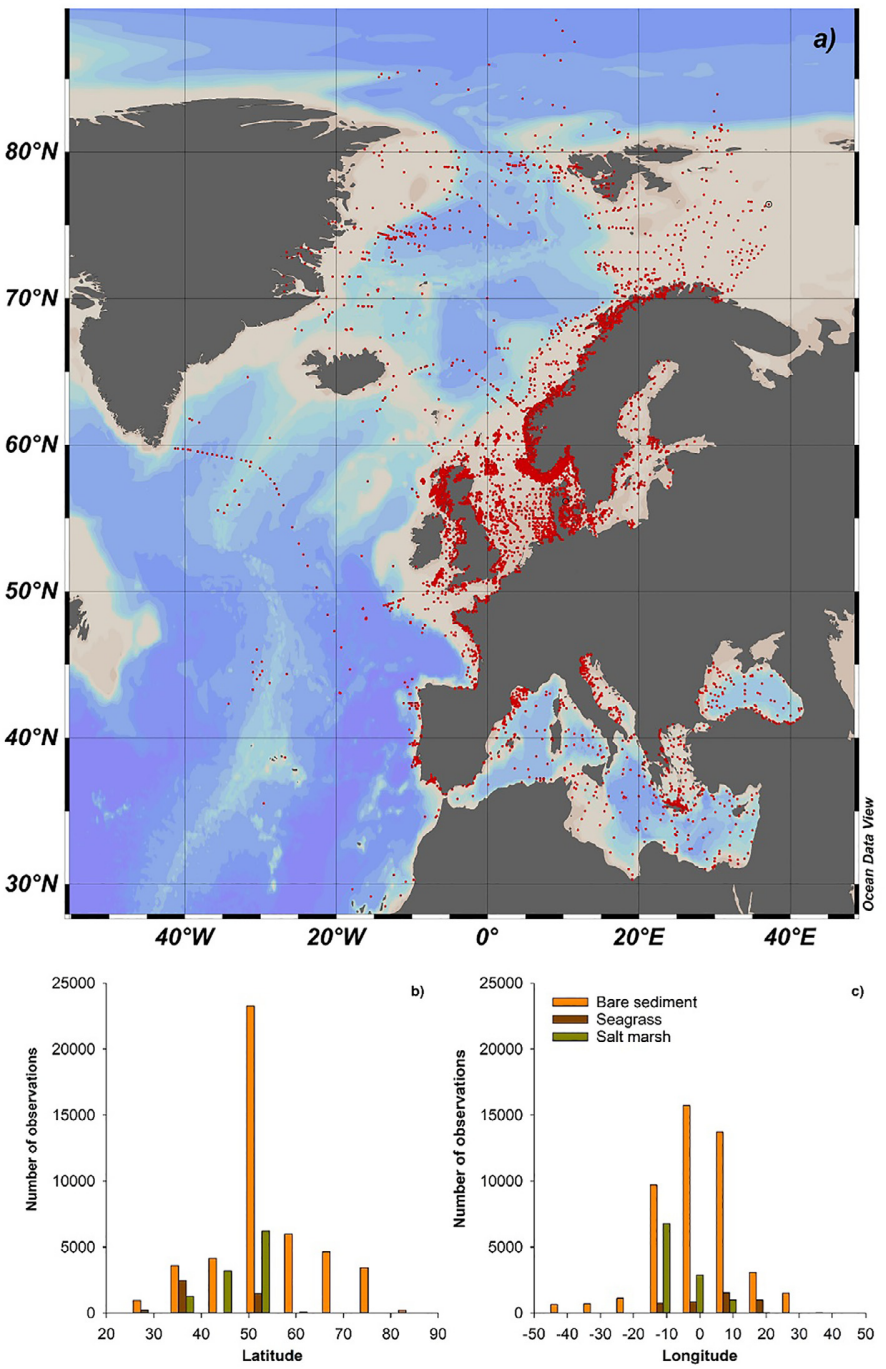
(a) Map showing the spatial distribution of the sediment organic carbon (%OC) data entries included in the EURO-CARBON database. Histograms show the number of %OC observations grouped into bins of 10° latitude (b) and longitude (c) for bare, seagrass and salt marsh sediments. Data from macroalgae habitats were not included in (b) and (c) as these data were collected in one location only.

The data included in EURO-CARBON represent a wide range of locations with different geological, physical and biogeochemical settings (Fig. 2). Observations were unevenly distributed, with northern regions of European regional seas holding a larger share of the observations, especially in open ocean regions (Fig. 2). The Mediterranean Sea and Black Sea were under-sampled compared with the North Sea, Baltic Sea region and Norwegian coastal waters (Fig. 2). Also, generally sediment OC observations were better resolved along the coast than the open ocean, with exception being the coastlines in countries such as Iceland, Ireland, Latvia, Lithuania, Croatia, Montenegro and Albania (Fig. 2) and the open ocean is therefore generally poorly resolved.

## 4 Experimental Design, Materials and Methods

In this compilation effort, we restricted our data search to coastal and deep-sea settings within the European Regional Seas, which here includes the Baltic Sea, the Black Sea, the North-east Atlantic Ocean, and the Mediterranean Sea. The sediment data were obtained from three types of sources, i.e., directly from data contributors, from online databases, and from scientific papers and reports to capture as many datasets as possible dealing with sediment OC in European regional seas. In cases of overlap between data received from different databases or from scientific papers, we prioritised the original dataset. Initially, in April 2023, the research community was invited through a public call to contribute data to establish a EURO-CARBON database of OC and related variables in marine sediments (for further details see supplement material). Researchers were encouraged to submit previously published and unpublished data. For this purpose, we created a template that all contributors used. Secondly, OC data were retrieved from databases including marine sediment data (see full list in Table S1). Thirdly, a detailed search was performed in Google Scholar using the search terms “sediment carbon” OR “sediment organic matter” OR “Blue Carbon”, which yielded 17700 entries (April 2023). We then filtered the query by searching for relevant content in the title and abstract, resulting in a total of 1112 potentially relevant studies. Further searches were conducted in the reference lists of the identified studies. Additional studies were included from existing reviews on sediment OC and finally, we included data from MSc or PhD theses, and other published reports based on our knowledge of the research field (see further details in Supplementary Methodology). As our focus was on marine sediments, we did not specifically target salt marsh OC data but where available these were also included in the EURO-CARBON database. In a few instances, above and below-ground living biomass of the sediment-associated habitat (e.g. seagrass) have been included in the EURO-CARBON database. The data included were gathered using various sampling and sorting techniques. However, all samples were dried after collection and thereafter analysed for variables such as OC and TN content and/or stable isotopic ratios using the below-mentioned techniques. The final dataset was derived from data collected, analysed and processed by many laboratories. Key information on sampling sites, methods and analytical techniques were provided along with the data. The list of variables included in the EURO-CARBON sediment and biomass database is shown in Table 1. Please do note that not all variables were available for each data entry.

**Table 1:** List of variables included in the EURO-CARBON sediment and biomass database. Variables marked in bold were mandatory.

Variables included in EURO-CARBON	
Sediment database	Biomass database
• Habitat	• Habitat
• Sample ID	• Location name
• Location name, Station ID, Core ID	• Station ID
• Year of sampling	• Sample ID
• Month of sampling	• Year of sampling
• Day of sampling	• Month of sampling
• **Latitude of sampling location (decimal degrees, WGS84)**	• Day of sampling
**• Longitude of sampling location (decimal degrees, WGS84)**	• Latitude of sampling location (decimal degrees, WGS84)
• Water depth (m) at which sediment core was obtained	• Longitude of sampling location (decimal degrees, WGS84)
• Water temperature (°C)	• Water depth (m) at which sample was obtained
• Salinity	• Temperature (°C)
• **Depth interval of sample when compacted (cm)**	• Salinity
• Depth interval of sample when decompacted (cm)	• Frame area (m^2^)
• Sediment porosity (Volume water/total volume; %)	• Dominating plant species
• Sediment water content (mass of water/total mass; %)	• Type of biomass
• Sediment dry bulk density (g cm^−3^)	• Wet weight (g)
• Organic matter content (OM) (%)	• Dry weight (g)
• **OC (% (dry weight))**	• Biomass (g m^−2^)
• OC (g C cm^−3^ (dry weight))	• OC (% (dry weight))
• Carbon stable isotope (ẟ^13^C in ‰) ratio	• Total Nitrogen (TN) content (% (dry weight))
• Total nitrogen (TN) content (% (dry weight))	• Carbon stable isotope (ẟ13C in ‰) ratio
• Total nitrogen (TN) content (g N cm^−3^)	• Nitrogen stable isotope (ẟ15N in ‰) ratio
• Nitrogen stable isotope (ẟ^15^N in ‰) ratio	• Description of biomass collection
• Total phosphorus (TP) content (% (dry weight))	• Methods for how data were obtained
• Total phosphorus (TP) content (g P cm^−3^ (dry weight))	**•** Data originator
• Carbon Reactivity Index (CRI) (ranging from 0 (Organic matter is fully biodegradable) to 1 (Organic matter is non-biodegradable)	• Originator institution
• Core dating:	• Contact of data originator
o Mass accumulation rate (g cm^−2^ year^−1^)	• Publications
o Sediment accumulation rate (mm year^−1^)	
o Carbon accumulation rate (g C m^−2^ year^−1^)	
o Total ^210^Pb activity (Bq kg^−1^)	
o Excess ^210^Pb activity (Bq kg^−1^)	
o Supported ^210^Pb activity (Bq kg^−1^)	
o ^14^C age (years) and ^14^C material	
• Sediment grain size (<0.063 mm, 0.063-0.25 mm, 0.25-0.5 mm, 0.5-1 mm, >1 mm)	
• **Methods for how data were obtained as well as the sediment sampling device**	
**• Data originator**	
• Originator institution	
• Contact of data originator

**Table 2:** Descriptive statistics for the sediment percentage organic carbon (%OC) data included in EURO-CARBON. The minimum (Min), maximum (Max), average values (± standard deviation, SD), median, coefficient of variance (CV), variance and number of samples (N) are shown for all and surface only data for all habitats (**All habitats**), macroalgae (**Algae habitat**), bare (**Bare sediment**), seagrass (**Seagrass**) and salt marsh habitats (**Salt marsh**). We used the term “salt marsh” broadly to also include e.g. *Phragmites australis*.

		All habitats	Algae habitat	Organic carbon (in %)		Salt marsh
				Bare sediment	Seagrass	
Min	All data	< 0.1	0.43	< 0.1	< 0.1	< 0.1
	Surface only	< 0.1	-	< 0.1	< 0.1	0.02
Max	All data	41.56	5.66	22.10	23.27	41.56
	Surface only	39.48	-	18.9	23.27	39.48
Average ± SD	All data	2.47 ± 3.37	1.98 ± 1.23	1.88 ± 2.03	2.37 ± 3.06	5.01 ± 5.96
	Surface only	2.42 ± 3.20	-	2.03 ± 2.11	3.01 ± 3.88	9.29 ± 7.92
Median	All data	1.39	2.16	1.20	1.11	3.03
	Surface only	1.46	-	1.39	1.15	6.97
CV	All data	1.37	0.62	1.07	1.30	1.19
	Surface only	1.32	-	1.04	1.29	0.85
Variance	All data	11.38	1.50	4.11	9.37	35.50
	Surface only	10.22	-	4.46	15.07	62.73
N	All data	61306	19	46308	4233	10746
	Surface only	29118	-	26392	705	1480

The field and analytical procedures applied to collect the data included in EURO-CARBON varied depending on the research focus and demands as well as technical capability. Although the variability of techniques and strategies may, to some extent, have impacted the measured sediment variables, we assume that given the large amount of data, such effects will not affect overall patterns in the data. Sampling techniques used to collect sediment include a range of sediment corer types (piston corer, box corer etc.). Sample decompaction and, at greater water depths depressurization, can impact the intactness of the obtained sediment cores and thereby the results; decompaction information is added where available.

Following sampling, retrieved sediment were typically divided into fixed sections based on depth ranges relevant to the study focus. After core retrieval and sectioning, sediment physical properties (e.g. grain size, and density) were in some instances measured. These physical properties reflect the geological and physical environment of the collected sediment, which also influences the chemical and biological processes within the sediments. Sediment porosity, defined as the volume of water-filled void space in relation to the total volume, was calculated from the weight loss upon drying of a sediment core segment of known weight and volume. The water content of the sediment core segments was calculated as the mass loss after drying divided by the bulk mass. The grain size distribution is important as it describes the study site’s geological setting and geochemical conditions, and can be used to distinguish sediment transport mechanisms and determines the porosity, especially in fine-grained sediment where porosity is controlled by grain size and mineralogy [2]. Grain size is commonly measured using a particle size analyser, such as a Laser Granulometer.

Dry bulk density (DBD) is defined as the mass of the total dry sediment divided by the total sample volume. The DBD is used to obtain volume-based OC stocks, and is calculated by dividing the weight of the dried sediment by the total volume of the wet sample. Overall, the DBD varies from close to 1 (high porosity sediment) to > 2 (low porosity sediments) and it is commonly determined by sampling a known volume of sediment and drying the sediment to a constant weight.

The two most common methods used to determine sediment OC content rely on conversion of OC into CO_2_ using either wet chemical or high-temperature oxidation techniques. More detailed descriptions of the analytical steps and methods used for marine sediments can be found in previous studies [13]. Briefly, the wet oxidation technique uses chemicals (such as potassium dichromate and sulfuric acid) to convert OC into CO_2_, which is subsequently quantified. The high-temperature oxidation technique, as used in a CHN-analyser [13], uses a "flash combustion'' of sediment OC to CO_2_, which is then detected using an infrared gas analyser or thermal conductivity detector. Both the wet oxidation and high-temperature oxidation techniques rely upon the separation of organic from inorganic carbon forms for an accurate quantification of OC. Earlier studies achieved the separation of inorganic carbon and OC by heating the sample (above 1050°C). More recently, acidification has been used to remove carbonates [13], but caution is needed as adding too much acid can lead to particle dissolution and loss of OC. OC data obtained in high-carbonate sediments, such as mussel beds and shell sand, might have a higher analytical error due to the potentially incomplete removal of carbonates (e.g., ([13]).

Determining sediment total nitrogen (TN) content relies, as for OC, on either a wet chemical oxidation or high-temperature oxidation technique. In the wet chemical oxidation approach, both organic and inorganic nitrogen compounds are oxidised to inorganic nutrients which are subsequently quantified through a colorimetric method [14]. In the high-temperature combustion approach, TN concentrations are determined based on conversion into nitrogen oxides, which are then determined by chemiluminescent emission using a nitric oxide detector or using a thermal conductivity detector. The high-temperature technique generally measures the OC and TN content simultaneously on the same sample as the analysers are fitted in series on a CHN elemental analyser [13]. Phosphorus forms in marine sediments are redox-dependent. When oxic conditions prevail, substantial amounts of phosphorus are retained in the sediment through adsorption to iron oxides (e.g., ([15]). In contrast, when anoxic conditions are present, organic forms dominate. Therefore, in studies focusing on sediment phosphorus, often several different phosphorus forms are measured [15]. The total phosphorus (TP) data included in EURO-CARBON were generally determined by initially using a digestion (e.g., microwave or high temperature) and/or a chemical digestion step [15]. Thereafter, the total inorganic phosphorus concentrations can be determined.

The stable isotope ratios of carbon (δ^13^C) and nitrogen (δ^15^N) have frequently been used to determine the organic matter origin (e.g., marine vs. terrestrial origin; see [16]), and are commonly analysed by nuclear magnetic resonance spectroscopy (NMR) or isotope ratio mass spectrometry (IRMS), often coupled in series with a CHN elemental analyser [17].

To report sediment accumulation rates as well as the age of the sediment, two dating techniques are commonly considered: analysing the sediment content of lead-210 (^210^Pb) or Carbon-14 (^14^C). The ^210^Pb-dating is conducted using gamma or alpha spectrometry and the decay of excess ^210^Pb activity is used to determine the sediment accumulation rate [18]. This method can be used to determine sediment deposits that are up to approximately 100 years old. The ^14^C-dating technique is based on the fact that living organisms incorporate radioactive carbon from the environment. When they die, no new carbon is incorporated and the accumulated ^14^C starts to decay. Thus, the known half-life of the ^14^C isotope can be used together with the content to determine the time since the OC was produced [19]. The method can date organic materials up to around 50,000 years old [19].

The carbon reactivity index (CRI) ranges from zero (fully reactive) to 1 (not reactive) and has in some studies been used to distinguish OC fractions depending on their thermal lability, which is suggested to be indicative of OC biodegradability [20].

Compilation and open sharing of existing data for important biogeochemical variables are relevant for determining large-scale patterns and potential drivers of OC accumulation and storage. In addition, does the limited spatial extent of sediment data across the seafloor, for example, has hampered the assessment of OC storage and its sensitivity to fisheries and other anthropogenic pressures. Therefore data products such as EURO-Carbon are highly warranted by both the research and policy communities.

The EURO-CARBON database was established so that the data should be findable, freely accessible and reusable. In addition, it constitutes an open-source quality-controlled dataset which can facilitate further detailed analysis using, for example, statistical or modelling tools.

From this initial overview of data, we identified potential future efforts which could improve the usefulness of large datasets such as EURO-CARBON. Firstly, measuring additional ancillary data such as DBD and sediment nutrient (nitrogen and phosphorus) content are recommended in future studies. Such ancillary data are often missing in the present dataset but are not only important to provide context and understand the processes driving sediment OC content, but variables such as DBD are also essential for calculating precise volume-based OC stocks. Additionally, our compilation highlights a clear spatial and potential geographic bias, with limited sampling in some parts of the European regional seas. This data gap needs to be addressed to provide a more accurate understanding of what controls sediment OC contents over larger spatial and temporal scales. Furthermore, there is a clear need for broad temporal and spatial datasets, which could capture both natural variability and potential human impacts in specific locations. This is vital as humans are directly impacting sediment OC cycling and storage through a range of direct mechanisms such as trawling and dredging, and indirectly through climate-related changes. We also recommend regular inter-calibration exercises for methods used to collect standard sediment variables, such as DBD, OC and nutrient content. Such inter-calibration efforts could ensure that the obtained data is comparable across different studies and study regions.

## Limitations

Not applicable.

## Ethics Statement

The authors have read and follow the ethical requirements for publication in Data in Brief and confirming that the current work does not involve human subjects, animal experiments, or any data collected from social media platforms.

## Data Availability

ZenodoEURO-Carbon Full Dataset (Original data).

## Appendix Supplementary materials

Image, application 1
