# Supplementary material for: A marine and salt marsh sediment organic carbon database for European regional seas (EURO-CARBON)
Source: Data Brief. 2025 May 3;60:111595. doi: 10.1016/j.dib.2025.111595 (PMC12149566; doi:10.1016/j.dib.2025.111595)
Supplement: Supplementary file 1 [file mmc1.docx]

**Supplement material for article:**

**A marine and salt marsh sediment organic carbon database for European regional seas (EURO-CARBON)**

**Authors**: Anna Elizabeth Løvgren Graversen, Christian Lønborg, Anna Maria Addamo, Sidsel Gurholt Pedersen, Silvia Chemello, Irene Alejo, Eugenia T. Apostolaki, Maria E. Asplund, William E.N. Austin, Dimitar Berov, Daniela Berto, Mats Björk, Kirsty Black, Nikola Bobchev, Stefano Bonaglia, Gunhild Borgersen, Tjeerd Bouma, Mark J. Costello, Martin Dahl, Elena Diaz-Almela, Panagiotis D. Dimitriou, Carlos M. Duarte, Carmen Leiva Dueñas, Pavlos T. Efthymiadis, Ines Mazarrasa Elosegui, Maria Recio Espinosa, Helena L. Filipsson, Marcos Fontela,, Stein Fredriksenꝉ, Helene Frigstad, Karine Gagnon, Catalina Andrea Garcia-Escudero,, Michele Giani, Anne Grouhel-Pellouin, Roberta Guerra, Martin Gullström, Hege Gundersen, Kasper Hancke, Claudia Majtényi-Hill, Corallie Hunt, Karina Inostroza, Ioannis Karakassis, Ventzislav Karamfilov, Stefania Klayn, Katarzyna Koziorowska, Karol Kuliński, Paul Lavery, Wytze K. Lenstra, Ana I. Lillebø, Ella Logemann, Paolo Magni, Núria Marbà, Candela Marco-Mendez, Marcio Martins, Miguel Angel Mateo, Briac Monnier, Peter Mueller, Joao M. Neto, Nafsika Papageorgiou, Carlos Eduardo de Rezende, Juan Carlos Farias Pardo, Jose Antonio Juanes De La Peña, Gérard Pergent, Nerea Piñeiro-Juncal, Joanne Preston, Federico Rampazzo, Gloria Reithmaier, Thorsten Reusch, Sarah Reynolds, Aurora M. Ricart, Rui Santos, Carmen B. de los Santos, Isaac R. Santos, Eduard Serrano, Oscar Serrano, Caroline P. Slomp, Craig Smeaton, Montserrat Sole, Ana I. Sousa, Timo Spiegel, Angela Stevenson, Jonas Thormar, Hilde Cecilie Trannum, Niels A.G.M. van Helmond, Sarah Paradis, Salvatrice Vizzini, Emma A. Ward, Yvonne Y. Y. Yau, Rym Zakhama-Sraieb, Imen Zribi, Olga M. Zygadlowska, Dorte Krause Jensen

**Supplementary Information Content:**

**Supplementary Methodology SM**

Supplementary Tables (S1-S4)

Supplementary Figure (S1)

**Methodology SM.**

The sediment data were obtained from three types of sources, i.e. directly from authors of previously published studies, from online databases, and from scientific papers to capture as many datasets as possible dealing with sediment organic carbon (OC) in European regional seas. In cases of overlap between data received from different databases or from scientific papers, we prioritised the original dataset.

Initially, in April 2023, the research community was invited through a public call (https://mpa-europe.eu/contribute-to-the-euro-carbon-database/) to contribute data to establish an EURO-CARBON database of OC and related variables in marine sediments. Researchers were encouraged to submit previously published and unpublished data. For this purpose, we created a template that all contributors used to submit the data. After five months (by August 2023) we have received data from 80 contributors, obtaining a dataset with 33650 entries over 5156 locations distributed across 19 Eu countries, 11 non-eu countries and high seas (https://mpa-europe.eu/the-euro-blue-carbon-database-at-first-glance/).

Secondly, OC data was also retrieved from all the databases including marine sediment data (see list in Table S1). We have scanned 60 national, regional or global databases, and obtained 65500 entries.

Thirdly, a detailed search was performed in Google Scholar using the search terms “sediment carbon” OR “sediment organic matter” OR “Blue Carbon”, which yielded 17700 entries (April 2023). We then filtered the query by searching for relevant content in the title and abstract, resulting in a total of 1112 potentially relevant studies.

**Table S1.** Overview of the databases that were searched for available sediment total organic carbon (OC) data. Please note that many of these databases are continuously updated and may not be fully reflected in EURO-CARBON (last access: 31 August 2023); e.g. the current updated version of the “Coastal Carbon Atlas” is not reflected in EURO-CARBON.

| Region or Country | Database name | Link |
| --- | --- | --- |
| Arctic region | Bolin Centre Database | https://bolin.su.se/data/ |
| Baltic region | HELCOM | [https://metadata.helcom.fi](https://metadata.helcom.fi/) |
| Baltic region | IOW | [www.io-warnemuende.de/data-portal.html](http://www.io-warnemuende.de/data-portal.html) |
| Belgium | Flanders Marine Institute (vliz) | <https://www.vliz.be/en/find-datasets> |
| Denmark | Danish monitoring data | [https://odaforalle.au.dk](https://odaforalle.au.dk/) |
| Denmark | GEUS | <https://eng.geus.dk/> |
| Finland | Finnish geological survey | <https://hakku.gtk.fi/> |
| Finland | SYKE | <https://ckan.ymparisto.fi/en/> |
| France | France geological survey | <https://www.brgm.fr/en> |
| France | Ifremer | <http://en.data.ifremer.fr/> |
| Germany | Bonick Eck | [https://www.bokniseck.de//database-access](https://www.bokniseck.de/database-access) |
| Germany | MUDABA | <https://geoportal.bafg.de/MUDABAnwendung/> |
| Germany | EU-SEASED (GEO SEAS) | <https://www.geo-seas.eu/search> |
| Germany | Marum | <https://www.marum.de/en/> |
| Germany | ZENODO | <https://zenodo.org/> |
| Germany/Netherlands | WALTER WADDEN | <https://datahuiswadden.waddenzee.nl/walter-dashboard/> |
| Great Britain | British Ocean Sediment Core Research Facility | <https://boscorf.org/our-services/data-requests> |
| Great Britain | Marine Scotland data | <https://data.marine.gov.scot/> |
| Great Britain | UK geological survey | [https://www.data.gov.uk](https://www.data.gov.uk/) |
| Great Britain | NDMC | <https://nmdc.no/en/havforskningsinstituttet/new/prosjekter/nmdc/datasets> |
| Great Britain | UK CEH | <https://catalogue.ceh.ac.uk/> |
| Great Britain | BODC | [www.bodc.ac.uk](http://www.bodc.ac.uk/) |
| Great Britain | CEFAS | <https://data.cefas.co.uk/> |
| Italy | NODC Italian Oceanographic Data Centre | <https://nodc.ogs.it/> |
| Netherlands | Dutch geological survey | <https://www.nlog.nl/en/core-collection-geological-survey> |
| Netherlands | Scheldt monitor | <https://www.scheldemonitor.org/dataproducts/> |
| Norway | Norwegian Environmental agency | [www.miljodirektoratet.no](http://www.miljodirektoratet.no/) |
| Norway | Norsk Marint data | <http://prod1.nmdc.no/UserInterface/#/> |
| Norway | AquaMonitor | <https://aquamonitor.niva.no/portal/> |
| Norway | Mareano | [www.mareano.no](http://www.mareano.no/) |
| Spain | CSIC-Spain | [http://data.utm.csic.es](http://data.utm.csic.es/) |
| Sweden | Sharkweb | <https://sharkweb.smhi.se/> |
| Sweden | Swedish Geological Survey | <https://apps.sgu.se/> |
| Whole region | ICES | <https://www.ices.dk/data/dataset-collections/Pages/default.aspx> |
| Whole region | Pangaea | [www.pangaea.de](http://www.pangaea.de/) |
| Whole region | Coastal carbon atlas | <https://ccrcn.shinyapps.io/CoastalCarbonAtlas/> |
| Whole region | Copernicus marine data | <https://marine.copernicus.eu/access-data> |
| Whole region | Dataset EEA | <https://www.eea.europa.eu/data-and-maps/data/> |
| Whole region | EMODNET | <https://emodnet.ec.europa.eu/> |
| Whole region | EUNIS | <https://eunis.eea.europa.eu/> |
| Whole region | GEOMAR | <https://data.geomar.de/> |
| Whole region | International ocean discovery program | https://web.iodp.tamu.edu/ |
| Whole region | MOSAIC | <http://mosaic.ethz.ch/> |
| Whole region | Wise marine | <https://water.europa.eu/marine/> |
| Whole region | Data Portal German Marine Research | [www.marine-data.de](http://www.marine-data.de/) |
| Whole region | Marine Geoscience Data System | <https://www.marine-geo.org/tools/> |
| Whole region | OBIS | <https://obis.org/> |
| Whole region | SEANOE | <https://www.seanoe.org/> |
| Whole region | Mendeley data | <https://data.mendeley.com/> |
| Whole region | SeaDataNet | <https://cdi.seadatanet.org/search> |

**Table S2**. The minimum (Min), maximum (Max), average (Avg.) (± standard deviation, SD), median, coefficient of variance (CV), variance and number of samples (N) are shown for sediment porosity, water content, dry bulk density, organic matter content, organic carbon (OC) density, delta 13 carbon isotopic ratios (δ^13^C; ratio of the two stable isotopes of carbon), nitrogen content, nitrogen density, delta 15 nitrogen isotopic ratios (δ^15^N; ratio of the two stable isotopes of nitrogen) and phosphorus content. Observations of OC density included both data directly reported, and values calculated where OC-content and dry bulk density were reported.

|  | **Porosity** | **Water content** | **Dry bulk density** | **Organic matter** | **OC-density** | **δ^13^C** | **Nitrogen** | **Nitrogen-density** | **δ^15^N** | **Phosphorus** |
| --- | --- | --- | --- | --- | --- | --- | --- | --- | --- | --- |
|  | (%) | (%) | (g cm^-3^) | (%) | (g C cm^-3^) | (‰) | (%) | (g N cm^-3^) | (‰) | (%) |
| Min | 7.0 | 8.4 | 0.01 | 0.01 | < 0.01 | -45.8 | < 0.01 | < 0.01 | -20.38 | 0.0008 |
| Max | 98.5 | 98.9 | 3.23 | 76.7 | 0.350 | -4.0 | 3.03 | 0.0041 | 18.97 | 0.5000 |
| Avg. ± SD | 64.7 ± 18.8 | 47.8 ± 20.7 | 0.83 ± 0.44 | 8.7 ± 9.6 | 0.019 ± 0.021 | -21.9 ± 3.9 | 0.24 ± 0.31 | 0.0011 ± 0.0008 | 5.53 ± 2.84 | 0.071 ± 0.051 |
| Median | 70 | 43.2 | 0.76 | 5.7 | 0.014 | -22.9 | 0.12 | 0.0008 | 5.62 | 0.0531 |
| CV | 0.3 | 0.4 | 0.53 | 1.1 | 1.080 | -0.2 | 1.27 | 0.6792 | 0.51 | 0.7247 |
| Variance | 353 | 428 | 0.19 | 92.5 | < 0.01 | 15.4 | 0.10 | 0.0000 | 8.05 | 0.0026 |
| N | 2687 | 5849 | 18821 | 3049 | 18373 | 7379 | 12640 | 1617 | 3745 | 918 |

**Table S3**. The minimum (Min), maximum (Max), average (Avg.) (± standard deviation, SD), median, coefficient of variance (CV), variance and number of samples (N) are shown for sediment accumulation rates (SAR), mass accumulation rate (MAR), carbon accumulation rate (CAR), total radioactivity per unit dry weight for lead 210 (Total ^210^Pb), carbon 14 age (^14^C) and grain size distribution in different size classes (Mud (<0.063 mm), fine sands (0.063-0.25 mm), medium sands (0.25-0.5 mm), coarse sands (0.5-1 mm) and very coarse sands (>1 mm)).

|  | **SAR** | **MAR** | **CAR** | **Total ^210^Pb** | **^14^C** | **Mud (<0.063mm)** | **Fine sands (0.063-0.25 mm)** | **Medium sands (0.25-0.5 mm)** | **Coarse sands (0.5-1 mm)** | **Very coarse sands (>1 mm)** |
| --- | --- | --- | --- | --- | --- | --- | --- | --- | --- | --- |
|  | (mm yr^-1^) | (g cm^-2^ yr^-1^) | (g cm^-2^ yr^-1^) | (Bq kg^-1^) | (Years) | (%) | (%) | (%) | (%) | (%) |
| Min | <0.001 | <0.001 | <0.01 | <0.01 | <1 | <1 | <1 | <1 | <1 | <1 |
| Max | 19 | 4700 | 247.28 | 1001 | 5195 | 100 | 95 | 100 | 57.9 | 71.9 |
| Avg. ± SD | 2.56 ± 2.92 | 80.6 ± 424.0 | 39.24 ± 39.14 | 86.3 ± 112.0 | 1142 ± 1222 | 29 ± 33 | 52 ± 26 | 23 ± 20 | 7 ± 9 | 10 ± 13 |
| Median | 1.7 | 1.75 | 30.5 | 49 | 1065 | 10.7 | 57.9 | 18.2 | 3.8 | 4.3 |
| CV | 1.1 | 5.3 | 1.0 | 1.3 | 1.1 | 1.1 | 0.5 | 0.8 | 1.2 | 1.3 |
| Variance | 9 | 180057 | 1532 | 12537 | 1492441 | 1068 | 688 | 386 | 77 | 176 |
| N | 1449 | 2431 | 720 | 2195 | 99 | 7682 | 4923 | 4354 | 1159 | 556 |

**Table S4**. The minimum (Min), maximum (Max), average (Avg.) (± standard deviation, SD), median, coefficient of variance (CV), variance and number of samples (N) are shown for above and below biomass wet and dry weight, biomass, organic carbon (OC) content, isotopic ratios (δ^13^C; ratio of the two stable isotopes of carbon), nitrogen content and nitrogen isotopic ratios (δ^15^N; ratio of the two stable isotopes of nitrogen).

|  | **Wet weight** | **Dry weight** | **Biomass** | **OC** | **δ^13^C** | **Nitrogen** | **δ^15^N** |
| --- | --- | --- | --- | --- | --- | --- | --- |
|  | (g) | (g) | (g m^-2^) | (%) | (‰) | (%) | (‰) |
| Min | 0.03 | < 0.01 | < 0.01 | 0.18 | -32.5 | 0.01 | 0.02 |
| Max | 946 | 202 | 73300 | 88.40 | -5.3 | 5.04 | 20.31 |
| Avg. ± SD | 40.9 ± 74.2 | 9.2 ± 26.3 | 1081 ± 4290 | 24.3 ± 17.1 | -19.5 ± 7.7 | 0.99 ± 0.80 | 8.34 ± 4.08 |
| Median | 17 | 2 | 135.00 | 30.70 | -19.1 | 0.9 | 7.9 |
| CV | 2 | 3 | 3.97 | 0.70 | -0.4 | 0.8 | 0.5 |
| Variance | 5506 | 691 | 18409496 | 292 | 59.2 | 0.6 | 16.6 |
| N | 507 | 514 | 1693 | 984 | 584 | 901 | 556 |


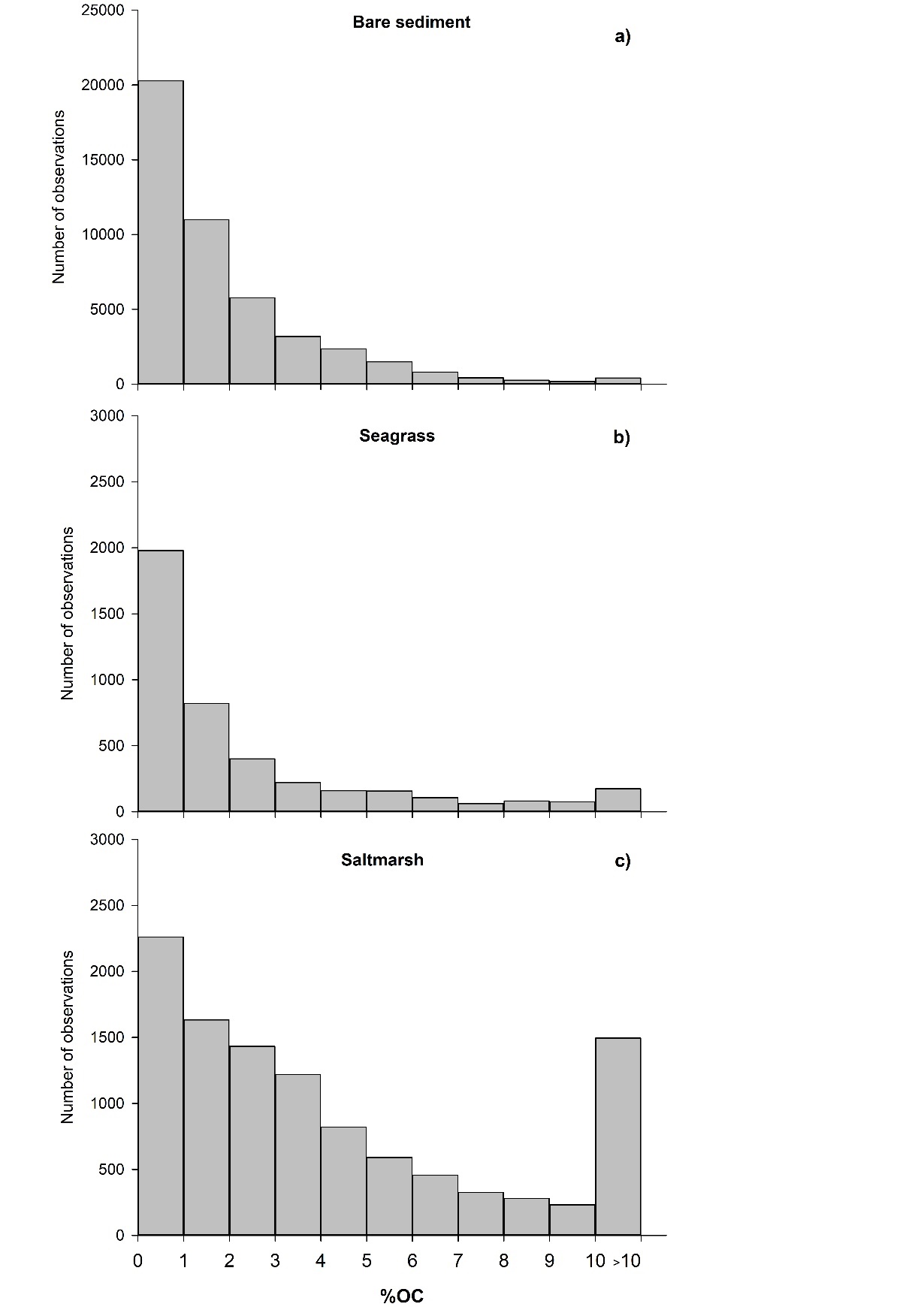


**Figure s1**. Histograms showing the distribution of the percentage of organic carbon (%OC) observations in (**a**) bare, (**b**) seagrass and (**c**) salt marsh sediments included in EURO-CARBON. Note that different y-axis ranges are used in the figures due to the large difference in number of observations. Macroalgae habitats were not included as these data were collected in a single location.
